# Predicting attitudes toward mitigation interventions and social distancing behaviors at the onset of the COVID-19 pandemic in the United States

**DOI:** 10.1080/21642850.2023.2247055

**Published:** 2023-08-16

**Authors:** Rachel E. Dinero, Nicole Shanguhyia, Rachel M. Hill, William Monti, Brittany L. Kmush

**Affiliations:** a Department of Psychological and Brain Sciences, Colgate University, Hamilton, NY, USA; b Department of Public Health, Syracuse University, Syracuse, NY, USA

**Keywords:** COVID-19, social distancing, personality, adult attachment, cultural orientation

## Abstract

**Aim:**

The goal of this research was to assess the influence of adult attachment, personality, and cultural orientation on social distancing and attitudes toward COVID-19 mitigation interventions.

**Methods:**

Survey data was collected across two samples (N_MTurk_ = 201, N_snowball_ = 242) in the US from April 29 to May 11, 2020. Adult attachment was assessed via the Experiences in Close Relationships Scale-Short Form (ECR-S; Wei, M., Russell, D. W., Mallinckrodt, B., & Vogel, D. L. (2007). The experiences in close relationship scale (ECR)-short form: Reliability, validity, and factor structure. *Journal of Personality Assessment*, *88*(2), 187–204), personality was assessed via the Ten Item Personality Inventory (TIPI; Gosling, S. D., Rentfrow, P. J., & Swann, W. B. (2003). A very brief measure of the Big-Five personality domains. *Journal of Research in Personality*, *37*(6), 504–528), cultural orientation was assessed via the Horizontal and Vertical Individualism and Collectivism Scale (Triandis, H. C., & Galfand, M. J. (1998). Converging measurement of horizontal and vertical individualism and collectivism. *Journal of Personality and Social Psychology*, *74*(1), 118–128), and social distancing and attitudes toward mitigation interventions were assessed via self-report measures developed for this assessment.

**Results:**

In the MTurk sample, agreeableness (*β* = .19) and conscientiousness (*β* = .26) predicted positive mitigation intervention attitudes. Agreeableness (*β* = .24) and vertical collectivism (*β* = .25) positively predicted social distancing, while attachment anxiety (*β* = −.32) and vertical individualism (*β* = −.32) negatively predicted social distancing. In our snowball sample, residing primarily in New York, openness (*β* = .18) and horizontal collectivism (*β* = .16) predicted positive intervention attitudes, while horizontal individualism (*β* = −.20) predicted negative attitudes. Social contact in this sample was low and not associated with predictor variables. In both samples, mitigation attitudes and social distancing were only moderately correlated.

**Implications:**

Our findings highlight the inherent inconsistency between attitudes and behaviors as well as the potential impact of mandated interventions on both attitudes and behavior.

Severe acute respiratory syndrome coronavirus 2 (SARS-CoV-2), the virus that causes coronavirus disease of 2019 (COVID-19) spread rapidly across the globe in early 2020. In the United States (US), local, state, and the national governments instituted many different non-pharmaceutical interventions aimed at slowing the spread of this novel virus. These preventative interventions included stay at home orders, limiting gathering sizes, wearing face masks, hygiene, and limited travel, among others. The goal of the present research is to assess the influence of individual differences in adult attachment, personality, and cultural orientation on attitudes toward COVID-19 interventions and adherence to these interventions, specifically social distancing. Below, we highlight the theoretical underpinnings of these individual differences and the existing research linking these characteristics to COVID-19 intervention attitudes and behaviors.

## Adult attachment anxiety and avoidance

Anxious attachment reflects the extent to which people are hyper-emotional and fearful of abandonment in close relationships. Attachment avoidance reflects the extent to which people are emotionally unavailable and uncomfortable with closeness (Brennan et al., 1998). In Israel, anxious attachment predicted higher levels of fear surrounding COVID-19 that, in turn, led to an increase in intervention adherence (Segal et al., 2021). Conversely, attachment avoidance predicted lower intervention adherence. Similarly, in an Italian sample, participants who were high in both attachment anxiety and external locus of control perceived higher risk from COVID-19, which increased the adoption of precautionary measures. In the same sample, attachment avoidance predicted lower perceived risk (Tagini et al., 2021). Further, Kafetsios (2022) found that group-level anxious attachment predicted lower initial number of COVID-19 cases across 53 countries, presumably owing to an uptick in preventive behaviors.

## Big five personality traits

The Big Five trait taxonomy is a framework for understanding individual differences that endure over time and across circumstances (John & Srivastava, 1999). Extraversion (i.e. the extent to which people are outgoing, active, and social) has been linked to difficulty in following COVID-19 social distancing interventions and higher stress levels (Carvalho et al., 2020; Gotz et al., 2021; Liu et al., 2021). Ohtsubo and Lyu (2021) found a marginally significant association between country-wide levels of extraversion and COVID-19 cases and deaths. Agreeableness (i.e. the extent to which individuals are likeable, cooperative, and trustful) was positively associated with adherence to self-isolation, social distancing, hand washing, and mask wearing (Willroth et al., 2021; Zajenkowski et al., 2020). Conscientiousness (i.e. the extent to which individuals are rule following, disciplined, thoughtful, and aware of others) predicted COVID-19 mitigation behaviors in Brazil, Qatari, and the US (Abdelrahman, 2022; Aschwanden et al., 2020; Carvalho et al., 2020). Neuroticism (i.e. the extent to which individuals demonstrate emotional instability, stress, anxiety, poor self-regulation, and increased threat perception) predicts increased perceived risk and stress surrounding COVID-19 (Aschwanden et al., 2020; Kroencke et al., 2020; Schneider, 2004), as well as increased handwashing (Abdelrahman, 2022; Liu et al., 2021). Openness (i.e. the extent to which individuals demonstrate creativity, imagination, and openness to new experiences and ideas) is linked to a decreased likelihood of underestimating health risks (Trobst et al., 2000), which has been found to decrease adherence to COVID-19 mitigation measures (Beca-Martinez et al., 2022).

## Cultural orientation

The model of individualism-collectivism originally developed by Hofstede (2001) is one of the most widely referenced models for cultural orientation (Bond, 2002; Hofstede, 2001; Hofstede & Hoppe, 2004). Cultural orientation can be further specified through the horizontal/vertical distinction (Gürhan-Canli & Maheswaran, 2000; Shavitt et al., 2006). Horizontal collectivist cultures emphasize interdependence and equality; cooperation and group cohesion are valued. Vertical collectivist cultures value interdependence with an acceptance of hierarchy. There is an emphasis on the importance of submission to ingroup authority and sacrifice. Horizontal individualist cultures value both independence and equality. While in vertical individualist cultures there is a drive for distinctiveness and status, and value is placed on competition and winning (Singelis et al., 1995).

In the early stages of the pandemic, communities with the highest rates of collectivism were the most likely to wear masks (Lu et al., 2021). Conversely, individualism was positively associated with COVID-19 mortality rates (Rajkumar, 2021). Horizontal individualism was positively associated with more frequent mask wearing and reduced rates of social interactions (Card, 2022; Chung et al., 2021). Horizontal collectivism was found to promote stronger trust in government authorities, and therefore, higher adherence to suggested interventions (Travaglino & Moon, 2021). Vertical individualism negatively predicted participant intention to engage in social distancing (Biddlestone et al., 2020), while vertical collectivism was associated with an increased likelihood to stay at home as a mitigation precaution (Card, 2022). Dinero et al. (2022) found that horizontal individualism predicted higher levels of risky social contact, while vertical collectivism predicted lower levels during the onset of the pandemic.

## The present research

The goal of the present study was to build on previous research linking individual differences in attitudes and behaviors to COVID-19 non-pharmaceutical interventions. Utilizing the Dinero et al. (2022) dataset mentioned above, we expand Dinero et al. (2022)’s findings linking cultural orientation to social distancing. Specifically, we present novel analysis predicting social distancing from adult attachment, personality traits, and cultural orientation. Moreover, we predict attitudes toward mitigation guidelines from these same variables. Additionally, we assess the association between intervention attitudes and behaviors. The inconsistency between attitudes and behaviors has been demonstrated in other contexts (Gross & Niman, 1975), but has yet to be addressed directly in the context of COVID-19 mitigation intervention.

Using this data, we hypothesized that positive intervention attitudes would be associated with adherence to social distancing interventions (i.e. lower levels of social contact). Further, we hypothesized that intervention attitudes would be positively predicted by attachment anxiety, agreeableness, conscientiousness, neuroticism, and vertical collectivism, and negatively predicted by attachment avoidance, and horizontal individualism. We also hypothesized that adherence to social distancing interventions would be positively predicted by attachment anxiety, agreeableness, conscientiousness, neuroticism, openness, and vertical collectivism, and negatively predicted by attachment avoidance, extraversion, and horizontal individualism.

## Methods

### Participants

A total of 443 individuals participated in an online survey administered from April 29 to May 11, 2020. There were 201 participants recruited through Amazon’s online participant recruitment platform Mechanical Turk. These participants were predominantly male [127(63%) males, 70(35%) females, 1(<1%) nonbinary, 3(1%) no gender response] and Caucasian [148(74%) Caucasian, 16(8%) Asian, 11(5%) Black/African American, 10(5%) Hispanic/Lantix, 4(2%) multi-ethnic, and 12(6%) no ethnicity response]. Participant age ranged from 21 to 69 years, with a mean of 38.04 years (*SD* = 11.63). MTurk participants were located across the US with participants from 40 states.^1^ A second snowball sample of 242 participants originated from a small private college in rural Central New York State. An email was sent to students and employees of the college, who were also encouraged to forward and post the survey link. Most of these participants were female [211(87%) female, 29(12%) male, and 2(<1%) nonbinary] and Caucasian [148(61%) Caucasian, 6(2%) Hispanic/Latinx, 1(<1%) Black/African American, 1(<1%) Persian, 9(4%) multi-ethnic, and 6(2%) no ethnicity response], and the age range was between 18 and 75 years, with a mean of 36.41 years (*SD* = 15.20). The majority of participants (78%) resided in New York, with the remaining participants from 23 states.^2^

### Measures

#### Demographic information

Demographic variables were obtained via participant self-report (i.e. age, gender, ethnicity, education, household income, zip code). Incidence rate of COVID-19 cases and population density were retrieved separately using the zip code reported by participants. Incidence rate was based on the number of new COVID-19 infections reported by zip code for the week of April 29, 2020 in the COVID-19 Data Repository by the Center for Systems Science and Engineering (CSSE) at Johns Hopkins University (Dong et al., 2020). The incidence rate value reflects the number of infections per 100,000 people in the zip code region. Population density information was retrieved by zip code from the most recent (2010) US Census (US Census Bureau, 2010). The population density variable was calculated by dividing zip code population by the square miles in the zip code.

#### Attitudes toward COVID mitigation interventions

Attitudes toward COVID-19 interventions were measured using a 9-item scale created for this study. Participants used a 5-point Likert scale to rate the importance of the nine behaviors recommended by the Center for Disease Control to slow or reduce the spread of COVID-19 at the time of the survey (i.e. washing hands; avoid touching eyes, nose, and mouth; using mask or cloth face cover; maintaining 6 feet of distance from others; not gathering in groups; not using public transportation; not attending small gatherings, not attending large gatherings, not going out in public). Participants were asked to rate, separately, the extent to which they perceived that these mitigation behaviors were important for people in the US to engage in to stop the spread of COVID-19 (population attitudes), were important for them personally to engage in (personal attitudes), and how practical these behaviors were for them personally to engage in (practical attitudes). We created scale scores for each type of attitude by averaging the items from each scale. These scales demonstrated adequate reliability (population attitudes, *α*_MTurk_ = .94, *α*_snowball_ = .90; personal attitudes, *α*_MTurk_ = .95, *α*_snowball_ = .91; practical attitudes, *α*_MTurk_ = .90, *α*_snowball_ = .82). Given the high intercorrelations between scales (Table 1), and for ease of regression analysis, we also created a general attitudes variable, by averaging all items from the three scales (*α*_MTurk_ = .97, *α*_snowball_ = .95).

**Table 1.** Mitigation Attitude Subscales and Contact Subscales Intercorrelations^1^.

	1.	2.	3.	4.	5.	6.
1. Population Attitudes	-	.94^a^	.86^a^	−.28^a^	−.30^a^	−.27^a^
2. Personal Attitudes	.88^a^	-	.86^a^	−.32^a^	−.34^a^	−.31^a^
3. Practical Attitudes	.65^a^	.72^a^	-	−.29^a^	−.30^a^	−.30^a^
4. Leisure Contact	−.26^a^	−.39^a^	−.30^a^	-	.95^a^	.91^a^
5. Essential Contact	−.18^a^	−.21^a^	−.25^a^	.21^a^	-	.92^a^
6. Work Contact	−.09	−.09	−.28^a^	.01	.19^a^	-

Notes.

^1^ MTurk sample on upper half of diagonal, snowball sample on lower half of diagonal

*p < .01

#### Adherence to social distancing interventions

To assess adherence to social distancing interventions, participants reported the frequency of social contact behaviors and activities in the past two weeks separately for work, non-work essential activities, and leisure activities. The frequency for each behavior was rated on a 5-point Likert scale (never, a few of the days, half of the days, most of the days, every day). The social behaviors of interest included: came within 6 feet of anyone who does not live with you, were you a part of a small gathering with people who do not live with you, were part of a large gathering of people who do not live with you, used public transportation, and used a taxi or rideshare service. This list of social behaviors was derived from CDC guidelines for recommended prevention behaviors at the time of the survey. The responses for the five behaviors were summed to form a work contact variable*,* an essential contact variable, and a leisure contact variable. All forms of contact were summed together to create a cumulative contact variable. While leisure, essential, and work contact were strongly intercorrelated in the MTurk sample, they were not in the snowball sample. This is likely a result of the lower variance in all social contact variables, discussed in in the next section.

#### Adult attachment

Adult attachment anxiety and avoidance were assessed using the Experiences in Close Relationships Scale-Short Form (ECR-S; Wei et al., 2007). Both attachment anxiety and attachment avoidance scales demonstrated adequate reliability (*α*_MTurk_ = .81, *α*_snowball_ = .76; *α*_MTurk_ = .78, *α*_snowball_ = .72, respectively).

#### Personality

We assessed the Big Five personality traits using the Ten Item Personality Inventory TIPI; (Gosling et al., 2003). The TIPI has been established as an appropriate measure of the Big Five personality traits (Gosling et al., 2003). Reliability coefficients for each trait are not reported as there are only two items per measure, and each are designed to tap into unique aspects of the trait (Gosling et al., 2003).

#### Cultural orientation

Cultural orientation was assessed using the Horizontal and Vertical Individualism and Collectivism Scale (Triandis & Galfand, 1998). Reliability coefficients across samples were adequate for horizontal collectivism (*α*_MTurk_ = .70, *α*_snowball_ = .57^3^), vertical collectivism (*α*_MTurk_ = .75, *α*_snowball_ = .64), horizontal individualism (*α*_MTurk_ = .76, *α*_snowball_ = .60), and vertical individualism (*α*_MTurk_ = .83, *α*_snowball_ = .67).

#### Ethics statement

All materials and procedures were evaluated as exempt by the Cazenovia College Institutional Review Board.

#### Statistical analysis

All statistical analysis was conducted in SPSS 28. For all correlations and regression models, we used a significance level of *p* < .05.

## Results

An a priori power analysis was conducted using G*Power3 (Faul et al., 2007) to estimate the required sample size for regression analysis predicting intervention attitudes/social distancing from attachment anxiety, attachment avoidance, controlling for social distancing/intervention attitudes respectively, age, gender, ethnicity, income, incidence rate, and population density. Based on the expected medium effect size (f^2^ = .15) and significance criteria (α = .05), a minimum sample size of 123 was required to achieve a power of .80.

### Sample comparisons

Given the demographic differences in the MTurk and snowball sample, we first assessed mean differences in all continuous demographic variables and constructs of interest (Table 2). Significant differences across samples were noted, specifically in population density and social contact. The differences in social contact across samples are not surprising given that the snowball sample was largely from New York State (78%), where social contact was impacted by mandatory restrictions on businesses, schools, and personal activities imposed by the state government during the time of the survey (Cuomo, 2020). Given these substantial differences, and that this separation would not violate sample sizes needed based on a priori power analysis, all subsequent analyses were conducted independently for each sample.

**Table 2.** Descriptive Statistics and Sample Comparisons.

	MTurk Sample	Snowball Sample	Sample Comparisons		
	Mean (SD)	Mean (SD)	Mean diff.	t-value	df
Population Attitudes	4.12(.82)	4.25(.65)	−0.13	−1.82^a^	436
Personal Attitudes	4.11(.92)	4.31(.72)	−0.20	−2.58^b^	436
Practical Attitudes	4.04(.80)	4.10(.65)	−0.06	−0.82	438
General Attitudes	4.09(.81)	4.22(.62)	−0.13	−1.91^a^	438
Leisure Contact	2.23(4.37)	0.74(1.07)	1.49	5.11^b^	435
Essential Contact	2.83(4.35)	1.15(1.02)	1.69	5.83^b^	438
Work Contact	2.57(4.54)	1.21(2.57)	1.36	3.93^b^	431
Cumulative Risk Contact	7.59(12.97)	3.11(3.22)	4.48	5.10^b^	423
Attachment Anxiety	2.63(1.19)	2.93(1.24)	−0.29	−2.47^b^	428
Attachment Avoidance	2.47(.94)	2.58(.84)	−0.11	−1.28	429
Extraversion	3.81(.94)	3.76(.84)	0.05	0.58	428
Agreeableness	4.01(.94)	4.16(.76)	−0.15	−1.80^a^	423
Conscientiousness	2.43(1.13)	2.71(1.03)	−0.28	−2.68^b^	425
Neuroticism	3.62(.95)	3.80(.84)	−0.18	−2.03^a^	427
Openness	2.88(.59)	2.97(.46)	−0.09	−1.82^a^	417
Horizontal Collectivism	3.54(.82)	3.58(.71)	−0.04	−0.51	424
Vertical Collectivism	3.35(.87)	3.69(.62)	−0.34	−4.66^b^	424
Horizontal Individualism	2.82 (1.06)	2.63(.76)	0.20	2.21^a^	424
Vertical Individualism	3.77 (.74)	4.09(.55)	−0.32	−5.07^b^	424
Demographic Variables					
Age	38.03(11.63)	36.41(15.20)	1.62	1.24^b^	440
Incidence Rate	0.16(.63)	0.13(.65)	0.03	0.53	422
Population Density	4883.95(13457.15)	1984.96(5400.68)	2898.99	3.03^b^	425

^a^ *p* < 0.05

^b^ *p* < 0.01

### Associations between intervention attitudes and social contact

We hypothesized that positive intervention attitudes would be associated with adherence to social distancing interventions (i.e. lower levels of social contact). This was supported by negative correlations between intervention attitudes and cumulative social contact in both the MTurk sample (*r *= −.32, *p* < .01) and the snowball sample (*r *= −.34, *p* < .01). While these correlations were significant, they were only moderate in size.

### Predicting intervention attitudes and adherence to social distancing interventions: mTurk sample

The primary goal of our analysis was to identify individual difference variables that were associated with positive intervention attitudes and social distancing. First, we ran correlations between the constructs of interest (Table 3). While cumulative contact and general attitudes were negatively correlated (*r* = −.32, *p* < .01), there were marked differences in the correlates of each. In the MTurk sample, general attitudes were negatively correlated with attachment anxiety (*r* = −.18, *p* < .05), attachment avoidance, (*r* = −.22, *p* < .05) and horizontal individualism (*r* = −.20, *p* < .05). General attitudes were positively correlated with extraversion (*r* = .24, *p* < .05), agreeableness (*r* = .27, *p* < .05), neuroticism (*r* = .14, *p* < .05), horizontal collectivism (*r* = .16, *p* < .05), vertical collectivism (*r* = .15, *p* < .05), and vertical individualism (*r* = .20, *p* < .05). Cumulative contact was positively correlated with attachment anxiety (*r* = .53, *p* < .05), attachment avoidance (*r *= .24, *p* < .05), conscientiousness (*r* = .19, *p* < .05), and horizontal individualism (*r* = .42, *p* < .05). Cumulative risk was negatively correlated with extraversion (*r* = −.39, *p* < .05), agreeableness (*r* = −.41, *p* < .05), neuroticism (*r* = −.25, *p* < .05), and vertical collectivism (*r* = −.33, *p* < .05).

**Table 3.** Correlations between Individual Difference Variables and Mitigation Attitudes/Social Contact: (MTurk/Snowball) Sample.

	Attitudes				Contact			
	Population	Personal	Practical	General	Leisure	Essential	Work	Cumulative
Attachment Anxiety	−.14^a^/.02	−.21^a^/.02	−.17^a^/−.11	−.18^a^/−.03	.54^a^/−.04	.53^a^/.19^a^	.50^a^/.06	.53^a^/.10
Attachment Avoidance	−.21^a^/−.15	−.24^a^/−.12	−.18^a^/−.16^a^	−.22^a^/−.16^a^	.23^a^/−.01	.26^a^/.05	.24^a^/.06	.24^a^/.06
Extraversion	.23^a^/.18^a^	.26^a^/.13	.19^a^/.15^a^	.24^a^/.16^a^	−.38^a^/.02	−.41^a^/.05	−.39^a^/−.01	−.39^a^/.01
Agreeableness	.24^a^/−.05	.27^a^/.00	.24^a^/.01	.27^a^/−.01	−.42^a^/−.04	−.42^a^/.02	−.39^a^/−.10	−.41^a^/−.09
Conscientiousness	−.03/.10	−.05/.11	−.04/−.02	−.04/.07	.20^a^/−.08	.22^a^/.08	.19^a^/.04	.19^a^/.03
Neuroticism	.12/.03	.13/.03	.15^a^/.07	.14^a^/.04	−.24^a^/.10	−.25^a^/.06	−.26^a^/−.03	−.25^a^/.02
Openness	.02/.21^a^	.01/.22^a^	−.01/.04	.01/.17^a^	.09/−.07	.12/−.08	.13/−.04	.11/−.09
Horizontal Collectivism	.16^a^/.06	.17^a^/.09	.13/.18^a^	.16^a^/.12	.05/−.08	.02/−.05	.03/−.05	.02/−.08
Vertical Collectivism	.15^a^/.17^a^	.15^a^/.15^a^	.12/.12	.15^a^/.15^a^	−.33^a^/−.07	−.34^a^/.02	−.32^a^/−.01	−.33^a^/−.02
Horizontal Individualism	−.18^a^/−.25	−.21^a^/−.23^a^	−.18^a^/−.26^a^	−.20^a^/−.27^a^	.41^a^/.18^a^	.42^a^/.10	.42^a^/.02	.42^a^/.10
Vertical Individualism	.19^a^/.07	.18^a^/.06	.20^a^/.09	.20^a^/.08	.01/.10	−.01/.06	−.05/−.00	−.03/.05

Notes.

^a^ *p* < 0.05

#### Predicting intervention attitudes

To assess the unique influence of predictor variables on general attitudes, we ran a linear regression predicting general attitudes from attachment anxiety, attachment avoidance, extraversion, agreeableness, conscientiousness, neuroticism, openness, horizontal collectivism, vertical collectivism, horizontal individualism, and vertical individualism (Table 4). The model fit the MTurk sample adequately (*R^2^* = .17, *adjusted R^2^* = .11, *F*(11,180) = 3.24, *p* < .001). We then ran the same model controlling for cumulative contact, age, gender, ethnicity, income, incidence rate, and population density (*R^2^* = 0.21, adjusted *R^2^* = 0.10, *F*(22, 147) = 1.81, *p* < .05). In the adjusted model, only agreeableness and conscientious emerged as significant predictors of general adherence attitudes. While these associations were in the predicted direction, the overall percent of variance in adherence attitudes explained by this model is relatively low.

**Table 4.** Linear Regression Analysis Predicting General Intervention Attitudes and Cumulative Social Contact (Standardized Betas).

	MTurk Sample (*β*)				Snowball Sample (*β*)			
	Attitudes	Attitudes^1^	Contact	Contact^2^	Attitudes	Attitudes^3^	Contact	Contact^4^
Attachment Anxiety	−.07	−.03	.29^b^	.32^b^	−.08	.00	.12	.04
Attachment Avoidance	−.14	−.16	.01	−.03	−.07	−.04	.05	−.03
Extraversion	.12	.11	−.12	−.15	.10	.08	.02	.08
Agreeableness	.27^b^	.19^a^	−.31^b^	−.24^b^	.05	.04	−.10	−.07
Conscientiousness	.21^a^	.26^a^	−.10	−.09	.14	.10	−.03	−.04
Neuroticism	.06	.01	−.09	−.05	.01	.03	.02	.03
Openness	.14	.11	−.09	−.06	.22^b^	.18^a^	−.15	−.04
Horizontal Collectivism	.09	.09	−.02	.01	.18^a^	.16^a^	−.13	.05
Vertical Collectivism	−.11	−.12	−.20^a^	−.25^b^	.05	.06	−.05	−.05
Horizontal Individualism	−.11	−.12	.18^a^	.15	−.25^b^	−.20^b^	.11	.04
Vertical Individualism	.03	.07	.30^b^	.32^b^	−.05	−.03	.12	.07

Notes.

^1^ Adjusted regression model predicting general attitudes controlling for cumulative contact (*β *= −.20), age (*β* = −.01), gender (*β* = .07), ethnicity (all nonsignificant), income (*β* = .17*), incidence rate (*β* = .02*)*, and population density (*β* = −.03)

^2^ Adjusted regression model predicting cumulative contact controlling for general attitudes (*β* = −.13), age (*β* = .03), gender (*β* = −.07), ethnicity (all nonsignificant), income (*β* = .01), incidence rate (*β* *= *−.23*), and population density (*β* = .29*)

^3^ Adjusted regression model predicting general attitudes controlling for cumulative contact (*β* = −.36**), age (*β* = .11), gender (*β* = −.09), ethnicity (all nonsignificant), income (*β* = −.18*), incidence rate (*β* = .06), and population density (*β* = .07)

^4^ Adjusted regression model predicting cumulative contact controlling for general attitudes (*β* = −.38**), age (*β* = −.15*), gender (*β* = −.03), ethnicity (all nonsignificant), income (*β* = −.23**), incidence rate (*β* = −.09), and population density (*β* = .02)

^a^ *p* < 0.05

^b^ *p* < 0.01

#### Predicting adherence to social distancing

To assess the unique influence of predictor variables on social distancing (i.e. low social contact), we ran a linear regression predicting cumulative contact from attachment anxiety, attachment avoidance, extraversion, agreeableness, conscientiousness, neuroticism, openness, horizontal collectivism, vertical collectivism, horizontal individualism, and vertical individualism (Table 4). The model fit the data adequately (*R^2^* = 0.45, *adjusted R^2^* = 0.42, *F*(11,169) = 12.69, *p* < .0001). We then ran the same model controlling for general intervention attitudes, age, gender, ethnicity, income, incidence rate, and population density (*R^2^* = 0.48, *adjusted R^2^* = 0.40, *F*(22,147) = 6.07, *p* < .001). In the adjusted model, social contact was positively predicted by attachment anxiety and vertical individualism, and negatively predicted by agreeableness and vertical collectivism. Additionally, this model explained 40% of the variance in social contact behavior.

### Predicting intervention attitudes and social contact: snowball sample

As with the MTurk sample, we first ran correlations between the constructs of interest (Table 3). As with the MTurk sample, cumulative social contact and general intervention attitudes were negatively correlated (*r *= −.34, *p* < .01) and the pattern of correlates with predictor variables was different. General attitudes were positively correlated with extraversion (*r* = .16, *p* < .05), openness (*r* = .17, *p* < .05), and vertical collectivism (*r* = .15, *p* < .05), and negatively correlated with attachment avoidance (*r* = −.16, *p* < .05) and horizontal individualism (*r* = −.27, *p* < .05). Cumulative contact was not significantly correlated with any of the predictor variables. This was not surprising given the overall low social contact and small variation in contact risk of the snowball sample. It is also interesting to note the different pattern of correlations with intervention attitudes across the MTurk and snowball samples.

#### Predicting intervention attitudes

To assess the unique influence of personality, adult attachment, and cultural orientation on intervention attitudes, we ran the same linear regression model used with the MTurk sample, predicting general attitudes from attachment anxiety, attachment avoidance, extraversion, agreeableness, conscientiousness, neuroticism, openness, horizontal collectivism, vertical collectivism, horizontal individualism, and vertical individualism (Table 4). Both the unadjusted model and the model controlling for cumulative contact risk, age, gender, ethnicity, income, incidence rate, and population density fit the data adequately (*R^2^* = 0.17, *adjusted R^2^* = 0.12, *F*(11, 204) = 3.76, *p* < .001, adjusted model: *R^2^* = 0.32, *adjusted R^2^* = 0.24, *F*(22, 183) = 3.92, *p* < .001). In the adjusted model, openness and horizontal collectiveness positively predicted general attitudes, while horizontal individualism was a negative predictor. This model explained 24% of the variance in general interventional attitudes, which is higher than the MTurk sample model (10%). This is particularly interesting given that the variance in general attitudes was significantly less in the snowball samples (*SD* = .62) as compared to the MTurk sample (*SD* = .81; *F*(200,241) = 1.71, *p* < .001).

#### Predicting adherence to social distancing

Despite the lack of significant correlations between cumulative contact risk and the predictor variables, we ran the same linear regression model used with the MTurk sample. The first model did not fit the data (*R^2^* = 0.06, *adjusted R^2^* = 0.01, *F*(11,199) = 1.23, *p* = 0.27), while the adjusted model did (*R^2^* = 0.28, *adjusted R^2^* = 0.20, *F*(22,183) = 3.25, *p* < .001). This is not surprising given that the predictor variables were not significantly correlated with cumulative contact risk.

## Discussion

In both the MTurk and snowball samples we found only moderate correlations between mitigation intervention attitudes and adherence to social distancing interventions. In the MTurk sample, we found that agreeableness and conscientiousness both predicted positive attitudes toward mitigation interventions, but only agreeableness predicted adherence to social distancing interventions. Adherence to social distancing was additionally predicted by vertical collectivism, while attachment anxiety and vertical individualism negatively predicted social distancing. These findings were consistent with our predictions, apart from attachment anxiety, which we expected to be positively associated with social distancing. In the snowball sample, openness and horizontal collectivism predicted positive attitudes toward interventions, while horizontal individualism predicted negative attitudes. This was consistent with our predictions, but notably different from the pattern of attitude predictors in the MTurk sample. Additionally, a higher percentage of variance in intervention attitudes was explained in the snowball sample than the MTurk sample, despite lower variance in intervention attitudes in the snowball sample.

### Implications

Taken together, these findings illustrate a distinct difference between mitigation related attitudes and behaviors. We stress the importance for future research is measuring attitudes and behaviors as distinct constructs, and caution against making predictions about mitigation behavior from expressed attitudes. Additionally, the difference in findings across two samples in the US highlights the heterogeneous nature of mitigation attitudes and behaviors. The differences in our samples are particularly interesting as our regression models control for incidence rate and population density. This suggests that intervention implementation, as well as potential other differences within and between states, may impact both attitudinal and behavioral pandemic responses.

### Limitations

One limitation of this study is the use of self-reported behaviors, specifically retrospective reports. Although, we purposely restricted the recall of behaviors to the previous two weeks to reduce the impact of inaccurate recall. We also recognize the potential impact of social desirability on all self-report responses. Further, our sample size was limited and we relied on short form measures of adult attachment (Wei et al., 2007) and personality traits (Gosling et al., 2003). While these versions are adequate, full versions of these measures are available (Fraley et al., 2000; John & Srivastava, 1999) and recommended for future research. Additionally, although cultural orientation was assessed using a validated measure (Triandis & Galfand, 1998), the horizontal collectivism subscale demonstrated only moderate reliability.

### Conclusion

We demonstrate here that attitudes toward mitigation and mitigation behavior are not strongly correlated and that they are predicted by different constructs. Additionally, while cultural orientation, personality traits, and attachment style are associated with mitigation attitudes and behavior in predicted ways, the cumulative influence of these variables explain only a modest percent of variance in mitigation behavior and attitudes. Further, we highlight the importance of sample characteristics in pandemic research, and caution against generalizing across US samples.

## Data Availability

The data described in this article are openly available in the Open Science Framework at https://osf.io/yed57/?view_only = 357d6e3f1d484ad58e9e0bcc543ab3c2.

## Notes

1 California (26), Texas (17), Florida (16), Pennsylvania (13), Ohio (11), New York (9), Georgia (7), New Jersey (7), North Carolina (7), Washington (6), Arizona (5), Kentucky (5), Missouri (5), Illinois (4), Massachusetts (4), Michigan (4), Mississippi (4), Nevada (4), Oregon (4), Tennessee (4), Wisconsin (4), Colorado (3), Maryland (3), Minnesota (3), South Carolina (3), Utah (3), Arkansas (2), Indiana (2), Maine (2), Rhode Island (2), Virginia (2), Connecticut (1), Delaware (1), Hawaii (1), Iowa (1), Louisiana (1), New Hampshire (1), New Mexico (1), South Dakota (1), and Wyoming (1).

2 New York (189), California (6), Pennsylvania (5), Florida (4), Vermont (4), Arizona (3), Massachusetts (3), Washington (3), Colorado (2), Connecticut (2), Ohio (2), Tennessee (2), Illinois (1), Indiana (1), Kansas (1), Maryland (1), Michigan (1), Minnesota (1), North Carolina (1), Oklahoma (1), Oregon (1), South Carolina (1), Texas (1), and Virginia (1)

3 Cronbach’s alphas ranging from .50 to .70 are considered moderately reliable and adequate in behavioral science research (Hinton et al., 2014)

## References

[CIT0001] Abdelrahman, M. (2022). Personality traits, risk perception, and protective behaviors of Arab residents of Qatar during the COVID-19 pandemic. *International Journal of Mental Health and Addiction*, *20*(1), 237–248. 10.1007/s11469-020-00352-7 32837433 PMC7307935

[CIT0002] Aschwanden, D., Strickhouser, J. E., Sesker, A. A., Lee, J. H., Luchetti, M., Stephan, Y., Sutin, A. R., & Terracciano, A. (2020). Psychological and behavioural responses to Coronavirus Disease 2019: The role of personality. *European Journal of Personality*, 10.1002/per.2281 PMC7361622 32836766

[CIT0003] Beca-Martinez, M. T., Romay-Barja, M., Falcon-Romero, M., Rodriguez-Blazquez, C., Benito-Llanes, A., & Forjaz, M. J. (2022). Compliance with the main preventive measures of COVID-19 in Spain: The role of knowledge, attitudes, practices, and risk perception. *Transboundary and Emerging Diseases*, *69*(4), e871–e882. 10.1111/tbed.14364 34730277 PMC8661801

[CIT0004] Biddlestone, M., Green, R., & Douglas, K. M. (2020). Cultural orientation, power, belief in conspiracy theories, and intentions to reduce the spread of COVID-19. *British Journal of Social Psychology*, *59*(3), 663–673. 10.1111/bjso.12397 32592420 PMC7361833

[CIT0005] Bond, M. H. (2002). Reclaiming the individual from Hofstede's ecological analysis - A 20-year odyssey: Comment on Oyserman et al. (2002). *Psychological Bulletin*, *128*(1), 73–77. 10.1037/0033-2909.128.1.73 11843548

[CIT0006] Brennan, K. A., Clark, C. L., & Shaver, P. R. (1998). Self-report measurement of adult romantic attachment: An integrative overview. In J. A. Simpson, & W. S. Rholes (Eds.), *Attachment theory and close relationships* (pp. 46–76). Guilford Press.

[CIT0007] Card, K. G. (2022). Collectivism, individualism and COVID-19 prevention: A cross sectional study of personality, culture and behavior among Canadians. *Health Psychology and Behavioral Medicine*, *10*(1), 415–438. 10.1080/21642850.2022.2069571 35528715 PMC9067981

[CIT0008] Carvalho, L. D. F., Pianowski, G., & Gonçalves, A. P. (2020). Personality differences and COVID-19: Are extroversion and conscientiousness personality traits associated with engagement with containment measures? *Trends in Psychiatry and Psychotherapy*, *42*(2), 179–184. 10.1590/2237-6089-2020-0029 32294713

[CIT0009] Chung, J.-B., Kim, B.-J., & Kim, E.-S. (2021). Mask wearing behavior and COVID-19: Synthetic effects of individualism and collectivism in Korea. *Research Square*, 10.21203/rs.3.rs-777573/v1

[CIT0010] Cuomo, A. M. (2020). *Amid ongoing COVID-19 pandemic, governor cuomo announces ‘NYS on PAUSE’ extended until May 15* https://www.governor.ny.gov/news/amid-ongoing-covid-19-pandemic-governor-cuomo-announces-nys-pause-extended-until-may-15.

[CIT0011] Dinero, R., Pieczonka, A., & Kmush, B. L. (2022). The association of cultural orientation with adherence to social distancing behaviors during the early COVID-19 pandemic in the United States: A cross-sectional survey. *PLOS Global Public Health*, *2*(8), e0000866–e0000866. 10.1371/journal.pgph.0000866 36962816 PMC10021574

[CIT0012] Dong, E., Du, H., & Gardner, L. (2020). An interactive web-based dashboard to track COVID-19 in real time. *The Lancet Infectious Diseases*, *20*(5), 533–534. 10.1016/S1473-3099(20)30120-1 32087114 PMC7159018

[CIT0013] Faul, F., Erdfelder, E., Lang, A.-G., & Buchner, A. (2007). G*Power 3: A flexible statistical power analysis program for the social, behavioral, and biomedical sciences. *Behavior Research Methods*, *39*(2), 175–191. 10.3758/BF03193146 17695343

[CIT0014] Fraley, R. C., Waller, N. G., & Brennan, K. A. (2000). An item response theory analysis of self-report measures of adult attachment. *Journal of Personality and Social Psychology*, *78*(2), 350–365. 10.1037/0022-3514.78.2.350 10707340

[CIT0015] Gosling, S. D., Rentfrow, P. J., & Swann, W. B. (2003). A very brief measure of the Big-Five personality domains. *Journal of Research in Personality*, *37*(6), 504–528. 10.1016/S0092-6566(03)00046-1

[CIT0016] Gotz, F. M., Gvirtz, A., Galinsky, A. D., & Jachimowicz, J. M. (2021). How personality and policy predict pandemic behavior: Understanding sheltering-in-place in 54 countries at the onset of COVID-19. *American Psychologist*, *76*(1), 39–49. 10.1037/amp0000740 33475389

[CIT0017] Gross, S. J., & Niman, C. M. (1975). Attitude-Behavior Consistency: A Review. *Public Opinion Quarterly*, *39*(3), 358. 10.1086/268234

[CIT0018] Gürhan-Canli, Z., & Maheswaran, D. (2000). Cultural variations in country of origin effects. *Journal of Marketing Research*, *37*(3), 309–317. 10.1509/jmkr.37.3.309.18778

[CIT0019] Hinton, P. R., Brownlow, C., & McMurray, I. (2014). *SPSS explained*. Routledge, Taylor & Francis Group.

[CIT0020] Hofstede, G. (2001). *Culture’s Consequesces: Comparing values, behaviors, institutions, and organizations across nations* (2 ed.). Sage Publications.

[CIT0021] Hofstede, G., & Hoppe, M. H. (2004). Introduction: Geert Hofstede's Culture's consequences: International differences in work-related values. *The Academy of Management Executive*, *18*, 73–74.

[CIT0022] John, O. P., & Srivastava, S. (1999). The Big Five trait taxonomy: History, measurement, and theoretical perspectives. In L. A. Pervin, & O. P. John (Eds.), *Theory and research* (Vol. 2, pp. 102–138). Guilford Press.

[CIT0023] Kafetsios, K. (2022). Collective reactions to epidemic threat: Attachment and cultural orientations predict early COVID-19 infection and mortality rates and trajectories. *Social Psychological and Personality Science*, *13*(7), 1126–1137. 10.1177/19485506211053461

[CIT0024] Kroencke, L., Geukes, K., Utesch, T., Kuper, N., & Back, M. D. (2020). Neuroticism and emotional risk during the COVID-19 pandemic. *Journal of Research in Personality*, *89*, 104038. 10.1016/j.jrp.2020.104038 33071370 PMC7550263

[CIT0025] Liu, S., Lithopoulos, A., Zhang, C. Q., Garcia-Barrera, M. A., & Rhodes, R. E. (2021). Personality and perceived stress during COVID-19 pandemic: Testing the mediating role of perceived threat and efficacy. *Personality and Individual Differences*, *168*, 110351. 10.1016/j.paid.2020.110351 32863508 PMC7442020

[CIT0026] Lu, J. G., Jin, P., & English, A. S. (2021). Collectivism predicts mask use during COVID-19. *Proceedings of the National Academy of Sciences of the United States of America*, *118(23)*), 10.1073/pnas.2021793118 PMC8201834 34016707

[CIT0027] Ohtsubo, Y., & Lyu, F. (2021). Is country-level extraversion associated with the number of COVID-19 cases and deaths? *Letters on Evolutionary Behavioral Science*, *12*(2), 39–45. 10.5178/lebs.2021.88

[CIT0028] Rajkumar, R. P. (2021). The relationship between measures of individualism and collectivism and the impact of COVID-19 across nations. *Public Health Practice (Oxford)*, *2*, 100143. 10.1016/j.puhip.2021.100143 PMC8411834 34494009

[CIT0029] Schneider, T. R. (2004). The role of neuroticism on psychological and physiological stress responses. *Journal of Experimental Social Psychology*, *40*(6), 795–804. 10.1016/j.jesp.2004.04.005

[CIT0030] Segal, S., Sharabany, R., & Maaravi, Y. (2021). Policymakers as safe havens: The relationship between adult attachment style, COVID-19 fear, and regulation compliance. *Personality and Individual Differences*, *177*, 10.1016/j.paid.2021.110832 PMC7957337 33746323

[CIT0031] Shavitt, S., Zhang, J., Torelli, C. J., & Lalwani, A. K. (2006). Reflections on the meaning and structure of the horizontal/vertical distinction. *Journal of Consumer Psychology*, *16*(4), 357–362. 10.1207/s15327663jcp1604_7

[CIT0032] Singelis, T. M., Triandis, H. C., Bhawuk, D. P. S., & Gelfand, M. J. (1995). Horizontal and vertical dimensions of individualism and collectivism: A theoretical and measurement refinement. *Cross-Cultural Research*, *29*(3), 240–275. 10.1177/106939719502900302

[CIT0033] Tagini, S., Brugnera, A., Ferrucci, R., Mazzocco, K., Pievani, L., Priori, A., Ticozzi, N., Compare, A., Silani, V., Pravettoni, G., & Poletti, B. (2021). Attachment, personality and locus of control: Psychological determinants of risk perception and preventive behaviors for COVID-19. *Frontiers in Psychology*, *12*, 10.3389/fpsyg.2021.634012 PMC8299752 34305708

[CIT0034] Travaglino, G. A., & Moon, C. (2021). Compliance and self-reporting during the COVID-19 pandemic: A cross-cultural study of trust and self-conscious emotions in the United States, Italy, and South Korea. *Frontiers in Psychology*, *12*, 565845. 10.3389/fpsyg.2021.565845 33796038 PMC8007877

[CIT0035] Triandis, H. C., & Galfand, M. J. (1998). Converging measurement of horizontal and vertical individualism and collectivism. *Journal of Personality and Social Psychology*, *74*(1), 118–128. 10.1037/0022-3514.74.1.118

[CIT0036] Trobst, K. K., Wiggins, J. S., Costa, P. T., Jr., Herbst, J. H., McCrae, R. R., & Masters, H. L., 3rd. (2000). Personality psychology and problem behaviors: HIV risk and the five-factor model. *Journal of Personality*, *68*(6), 1233–1252. 10.1111/1467-6494.00133 11130739

[CIT0037] US Census Bureau. (2010). *Population, housing units, area and density*. https://data.census.gov/cedsci/.

[CIT0038] Wei, M., Russell, D. W., Mallinckrodt, B., & Vogel, D. L. (2007). The experiences in close relationship scale (ECR)-short form: Reliability, validity, and factor structure. *Journal of Personality Assessment*, *88*(2), 187–204. 10.1080/00223890701268041 17437384

[CIT0039] Willroth, E. C., Smith, A. M., Shallcross, A. J., Graham, E. K., Mroczek, D. K., & Ford, B. Q. (2021). The health behavior model of personality in the context of a public health crisis. *Psychosomatic Medicine*, *83*(4), 363–367. 10.1097/PSY.0000000000000937 33790198 PMC9011405

[CIT0040] Zajenkowski, M., Jonason, P. K., Leniarska, M., & Kozakiewicz, Z. (2020). Who complies with the restrictions to reduce the spread of COVID-19?: Personality and perceptions of the COVID-19 situation. *Personality and Individual Differences*, *166*, doi:10.1016/j.paid.2020.110199 PMC7296320 32565591

